# Guideline of the German Society of Neurology (DGN): "diagnosis and therapy of HIV-1-associated neurological disorders”

**DOI:** 10.1186/s42466-026-00519-y

**Published:** 2026-08-13

**Authors:** Katrin Hahn, Gabriele Arendt, Christian Eggers, Cristina Granziera, Matthias Maschke

**Affiliations:** 1 Department of Neurology with Experimental Neurology, Charité- Universitätsmedizin Berlin, Corporate Member of Freie Universität Berlin and Humboldt-Universität Zu Berlin, Charitéplatz 1, 10117 Berlin, Germany; 2 Neuro-Centrum Düsseldorf, Stadtbezirk, Germany; 3 Universitätsklinik for Neurologie, Kepler Universitätsklinikum Linz, Linz, Austria; 4 Department for Biomedicine, Research Center for Clinical Neuroimmunology and Neuroscience Basel, Universitätsspital Basel, Universität Basel, Basel, Switzerland; 5 Department of Neurology and Neurophysiology Krankenhaus, Barmherzigen Brüder Trier Campus Trier Medical University Mainz, Nordallee 1, 54292 Trier, Germany; 6 Klinik für Neurologie und Experimentelle Neurologie, Universitätsmedizin Charité Berlin Amyloidosis Center Charite Berlin (ACCB), Charitéplatz 1, 10117 Berlin, Germany

## Abstract

Epidemiology and treatment of HIV changed substantially within the last three decades. However, HIV-associated neurological disorders such as mild forms of HIV-associated neurocognitive disorder (HAND) as well as HIV-associated distal symmetrical sensory polyneuropathy are amongst the most frequent complications in patients with longstanding HIV infection. Opportunistic infections occur less frequently compared to former years, but especially in patients with late presentation of HIV progressive multifocal leukoencephalopathy (PML) (0.7 per 1,000 patient-years), toxoplasma encephalitis (0.4 per 1,000 patient-years) and cryptococcal meningitis (0.2 per 1,000 patient-years) remain the most common opportunistic infections. The guidelines for diagnosis and treatment of HIV-associated neurological disorders of the German Society of Neurology were revised addressing recent changes in treatment opportunities of HAND, HIV associated complications of the peripheral nervous system and muscles and opportunistic infections of the central nervous system (CNS).

## What´s new

In 2014, UNAIDS launched the 90-90-90 strategy, which aims to ensure that by 2030, 90% of all people living with HIV worldwide are diagnosed, are treated and achieve viral suppression. UNAIDS’s current AIDS Strategy 2021–2026, and the subsequent 2026–2031 strategy expands the targets to a 95-95-95 strategy.

In addition to new compounds within established treatment classes (e.g. the integrase inhibitor cabotegravir; the non-nucleoside reverse transcriptase inhibitor (NNRTI) doravirine) new treatment targets have been developed (monoclonal CD4 antibodies, capsid inhibitors, attachment inhibitors).

Despite the high efficacy of combination antiretroviral therapy (cART), mild forms of HIV-associated neurocognitive disorder (HAND) persist. This represents a therapeutic dilemma, which is likely to be resolved in the future only by development of new treatment strategies, such as the delivery of antiretroviral substances via nanoparticles, or strategies leading to the reactivation of latent provirus in the CNS followed by antiretroviral eradication [1, 2].

### The most important recommendations

- If HIV-1 infection is diagnosed, antiretroviral therapy is indicated regardless of the CD4 cell count and the clinical presentation.
- In patients with HIV-1-associated dementia (HAD) on antiretroviral therapy with suppressed plasma HIV-RNA levels, cerebrospinal fluid (CSF) analysis should be performed; if viral replication is detected, resistance testing should be carried out, followed by a switch to cART containing drugs with the greatest possible CSF penetration. CSF penetration is best documented for azidothymidine (AZT), abacavir (ABC), lamivudine (3TC), emtricitabine (FTC), nevirapine (NVP), darunavir (DRV), lopinavir (LPV/r), raltegravir (RAL), dolutegravir (DTG) and maraviroc (MVC).
- Polyneuropathy: Distal symmetrical sensory polyneuropathy is by far the most common peripheral manifestation associated with HIV infection. In cases of painful polyneuropathy with significant paralysis, vasculitis must be considered as a differential diagnosis, particularly if the clinical pattern is asymmetrical (perform a nerve/muscle biopsy).
- Sporadic inclusion body myositis (IBM) represents a rare muscular manifestation which is most likely a manifestation of T-cell dysregulation and impaired muscular repair. Patients with HIV-associated IBM are younger than those with classic IBM, have elevated CK levels and show greater involvement of the proximal muscles of the upper limbs. Approximately two-thirds of patients have antibodies against NT5C1A.
- With rapidly progressing central neurological deficits, an opportunistic infection should be considered. Progressive multifocal leukoencephalopathy (PML) (0.7 per 1,000 patient-years), toxoplasma encephalitis (0.4 per 1,000 patient-years) and cryptococcal meningitis (0.2 per 1,000 patient-years) are the most common opportunistic infections.
- Diagnosis of opportunistic infections: In cases where PML is the primary suspicion, JC virus PCR should only be performed in laboratories with experience in this technique. Cerebral cryptococcosis should be investigated using a stained smear of fresh (no more than one hour old) CSF or the latex antigen test.
- In patients with opportunistic infections that occur within a few months of starting successful cART, immunoreconstitution syndrome (IRIS) should be considered. This frequently presents as “unmasking IRIS” (symptoms of an opportunistic infection appear after the start of cART) or as “paradoxical IRIS” (recurrence of symptoms following an initial improvement under newly initiated cART).
- Many drugs with a neurological indication (e.g. antiepileptic drugs) interact pharmacokinetically with antiretroviral agents. Useful information can be found, for example, in the Liverpool HIV Drug Interaction Checker: http://www.hiv-druginteractions.org.
- In HIV-infected individuals with good immune status and white matter lesions suggesting radiological HIV encephalopathy on the MRI, may represent a CNS escape, regardless of the clinical and neurological findings; this involves the replication of HIV in the CNS independently of the systemic compartment [3, 4]. These patients must be closely monitored for the manifestation of cerebral, virus-associated diseases. As a differential diagnosis cerebrovascular disease must be ruled out, particularly in older age groups [5].
- Patients with Neuro-AIDS should be treated by neurologists who have experience with this condition.

## Introduction

10–20% of HIV-infected patients become symptomatic due to a neurological manifestation [6]. Primary HIV-associated neuropathological findings can affect all levels of the neural axis and occur at any stage of the infection. Individual clinical presentations such as HIV encephalitis or myelitis are manifestations of a productive viral infection of the nervous system, whilst others, such as vacuolar myelopathy or distal symmetrical polyneuropathy, appear to be multifactorial. The pathophysiological processes include immunological and metabolic dysregulation [7].

## Pathophysiology

HIV primarily infects CD4 + T cells, macrophages and dendritic cells. As a retrovirus, it has the ability to integrate its genome into the host, making eradication (to date) virtually impossible [8]. The virus exhibits a wide range of genetic diversity and a high mutation rate. This enables it to evade detection by the immune system and develop resistance to antiretroviral drugs [9].

HIV can be detected in the CSF as early as one week after primary infection [10, 11], with a higher viral load in patients with neurological symptoms [12]. CNS acts as a viral reservoir [13], with mononuclear phagocytes playing a key role in pathogenesis [14]. Active viral replication takes place in the microglia [15]. This is associated with neuroinflammation, which is observed histopathologically in both the early (e.g. aseptic leptomeningitis) and late stages of the disease (e.g. HIV dementia), as well as in neurologically asymptomatic HIV-infected individuals [7]. A systematic meta-analysis suggests that interleukin-1 beta and tumor necrosis factor-alpha play a significant role in the development of HIV dementia [16]. Viral CNS penetration is the subject of active research [17, 18] and will significantly influence future strategies for the eradication of HIV [1, 2].

## Definition and classification

### Aseptic leptomeningitis

Aseptic leptomeningitis is an early complication of HIV infection and typically occurs within the first two weeks of infection as a manifestation of acute, viral replication with high viremia [19]. Clinically, it cannot be distinguished from other aseptic meningoencephalitides.

### HIV-associated neurocognitive disorder (HAND)

Approximately 30–50% of all patients develop neurocognitive impairment in the course of HIV infection [20, 21]. The majority are mild, involving cognitive slowing, concentration difficulties and memory problems, as well as behavioral abnormalities [22]. The so-called Frascati scientific criteria describe three stages of HAND [23]. Although the criteria were primarily developed for scientific purposes and their application in everyday clinical practice is subject to controversy [24], their use is widespread and justified, particularly with the aim of establishing a standardised nomenclature.

First stage of virus-associated brain disease (= asymptomatic HIV-associated neuropsychological deficit, ANPD).

- Acquired deficits in at least two cognitive domains (verbal fluency, executive functions, information processing speed, attention, working memory, or verbal and visual learning); the results of at least two standardised tests must fall outside the range of one standard deviation.
- Impairments do not affect everyday life.
- Other causes of ANPD must be ruled out: e.g., severe depressive episodes, psychosis, or chronic drug and/or alcohol use.

The definition of ANPD is controversial due to the absence of subjective symptoms in patients and is largely the subject of scientific research. However, numerous studies have shown that patients with ANPD are at a higher risk of developing subsequent stages of HAND [25, 26].

Stage two of the virus-associated brain disorder (= mild HIV-associated neurocognitive disorder, MNCD).

- As with ANPD: the results of at least two standardised tests must fall outside the range of one standard deviation.
- These cognitive impairments are evident in everyday life: patients report a reduced mental alertness, inefficiency at work and in managing their own households, as well as difficulties in social interactions.
- The deficits listed under (a) must primarily be reported or confirmed by the patient’s family and/or partner.
- The deficits have persisted for more than one month.
- As with ANPD: other causes of MNCD must be ruled out.

Stage 3 of virus-associated brain disease (= HIV-associated dementia, HAD).

- Significant cognitive impairment in at least two psychometric tests across different areas of cognitive functioning; test results fall outside the range of two standard deviations.
- Daily life is severely impaired and, for the most part, cannot be managed without assistance.
- As with MNCD: the duration of the deficits is more than one month. Other causes of HAD must be ruled out.

If, following a diagnosis of ANPD or MNCD, the criteria are no longer met during a follow-up examination, the condition is referred to as ANPD/MNCD “in remission”; the diagnosis of “HAD”, however, is irreversible in this respect. In addition to the cognitive impairments described, patients with HAD usually also exhibit fine motor skills disorders and, in very advanced stages, spastic tetraparesis with bladder dysfunction and mutism [3].

Approximately 10% of patients with HAD suffers from epilepsy [27].

## Screening

Despite numerous efforts to develop screening tools, no consensus on the timing or the methodology has been reached [28]. Conflicting viewpoints reflect the questionable relevance in patients with an ANPD, limited screening capacities and, conversely, the potential treatability of early stages through CSF-penetrating antiretroviral drugs [29]. The HIV Dementia Scale was developed in 1995 with the aim of detecting patients with HAD [30]. For this patient group, a sensitivity of 80% and a specificity of 91% were reported [31–33]. Today, sensitivity and specificity are significantly lower due to changes in the clinical presentation of dementia under cART. Furthermore, identifying the first two stages of HAND is considerably more difficult. It is proposed that the cut-off score be adjusted from 10 to 11/12 [34] or, according to some authors, to 14/16 points [35, 36]. Alternative bedside tests, such as the MoCA (Montreal Cognitive Assessment) show low sensitivity [29].

## Risk factors for the development of HAND

Risk factors (RF) for the development of severe systemic HIV infection are also risk factors for the development of HAND. These include a low CD4 + nadir or a low current CD4 + count, the duration of HIV infection, a history of AIDS-defining conditions, and a high HIV viral load [29]. Age correlates positively with the development of HAND [37]. HIV-positive people over the age of 50 are twice as likely to develop HAND as younger HIV-positive individuals [38].

In addition, comorbidities that limit cognitive reserve capacity or cART adherence, irrespective of HIV, are relevant. These include: hepatitis C [20], vascular and metabolic risk factors [39], drug and/or alcohol abuse [40, 41] and psychiatric comorbidities, the latter of which are frequently associated with poor adherence to cART [42].

## HIV-1-associated myelopathy (HIVM) and HIV myelitis

### HIVM

HIVM is rare and occurs predominantly in late stages of infection. The most common histomorphological correlate is intramyelinic vacuolisation with an emphasis on the posterior and lateral columns, and the presence of lipid-laden macrophages [7].

The condition is characterised by slowly progressive lower-limb-predominant tetraparesis and a spastic-ataxic gait, hyperreflexia and pyramidal tract signs, sphincter dysfunction, and glove-and-sock-distribution sensory disturbances without a clearly defined level. In 60% of patients with HIVM, HIV-associated dementia is also present [43].

### HIV-myelitis

HIV myelitis is a rare complication of advanced HIV infection that occurs independently of cerebral involvement. In a series of autopsies of untreated HIV-infected individuals, 8% were affected [44]. The histopathology is comparable to HIV encephalitis and characterised by inflammatory microglial nodular pathology.

## Neuromuscular complications

Neuromuscular complications of HIV infection are common and can occur at any stage. The spectrum is diverse and includes immunological, infectious, vasculitic, toxic and neoplastic processes. Among the etiologies, HIV-associated distal symmetric polyneuropathy (HIV-DSP) is clinically predominant. Prevalence rates of up to 35% were reported prior to the cART era [45, 46].

## HIV-1-associated neuropathies

### HIV-DSP

HIV-associated DSP is a typical length-dependent polyneuropathy (PNP) characterised primarily by sensory symptoms and, at most, mild distal paralysis in advanced stages. Asymptomatic patients are also observed, who are only detected during neurological examination or electrophysiological testing [47]. The pooled prevalence is 29% for antiretroviral-naive patients and rises to 38% for patients regardless of the stage of the disease [48].

Risk factors include advanced age, the concurrent presence of diabetes mellitus or hypertriglyceridaemia, or substance abuse [49–51]. From a pathophysiological perspective, there is evidence of inflammation in dorsal spinal ganglion cells, leading to secondary neuronal damage [52].

Antiretroviral agents, particularly those from the group of nucleoside analogue reverse transcriptase inhibitors (NRTIs) such as didanosine (ddI), stavudine (d4T) and zalcitabine (ddC), can cause a sensitive polyneuropathy known as antiretroviral toxic neuropathy (HIV-ATN), which is phenotypically indistinguishable from HIV-DSP [53, 54]. Due to their high toxicity, these substances are no longer used worldwide.

### Acute inflammatory demyelinating polyradiculoneuritis

The condition is also known as HIV-1-associated Guillain-Barré syndrome (GBS) and may occurs in the early stages of HIV infection, sometimes in the context of seroconversion [55]. Clinically, the presentation does not differ from the non-HIV-associated forms. However, relapses or progression to chronic inflammatory demyelinating polyradiculoneuropathy (CIDP) are more common [56].

### Chronic inflammatory demyelinating polyradiculoneuropathy (CIDP)

HIV-associated CIDP is rare and occurs mainly in patients with mild to moderate immunodeficiency. Clinically, its presentation is no different from that of non-HIV-associated forms.

### HIV-1-associated vasculitic polyneuropathy

This clinical presentation should be considered in the differential diagnosis, particularly in patients with subacute or markedly progressive asymmetric polyneuropathy presenting with a focal pattern and relevant paresis.

### Polyneuropathy in diffuse infiltrative lymphocytosis syndrome (DILS)

The condition is rare and occurs in the early stages of infection. It is an HIV-associated autoimmune complication characterised by CD8 lymphocytosis and involving multiple organ infiltration (e.g. lungs, kidneys, liver). The presentation shows parallels to Sjögren’s syndrome, with enlargement of the parotid gland (sometimes involving the facial nerve) and xerostomia [57]. In rare cases, there is meningeal involvement in the form of aseptic leptomeningitis with involvement of the lumbosacral plexus fibres. Histopathologically, there is evidence of endo- and epineural CD8 T-cell infiltration as well as multiple monocytes and macrophages in which HIV antigen can be detected [58].

*Mononeuropathies* *(e.g. facial nerve palsy)* can occur at any stage of the disease.

*Polyradiculoneuritis* caused by opportunistic pathogens (< 1%) usually occurs in the AIDS stage and is frequently caused by CMV.

## HIV-1-associated myopathies

At the time of seroconversion, non-specific myalgia that resolves over time is frequently observed. In the context of HIV infection, various inflammatory myopathies (e.g. polymyositis, inclusion body myositis) or antiretroviral-induced (usually zidovudine) myopathies may occur; in the AIDS stage, opportunistic infections with muscular manifestations may also arise. Diagnostic classification is usually based on histopathological criteria [59].

### Antiretroviral-induced myopathies

Zidovudine-associated myopathy is well documented as a manifestation of mitochondrial damage. The risk increases with cumulative dose [60]. Clinically, it presents as myalgia predominantly affecting the proximal muscles, with or without paresis, and in some cases also atrophy and fatigue [61]. Its clinical relevance is negligible, as zidovudine is rarely used anymore.

### Inflammatory myopathies

A rare manifestation is sporadic inclusion body myositis, which reflects T-cell dysregulation and is associated with impaired muscle repair processes [62, 63]. Compared with the non-HIV-associated variant, the age of onset is significantly lower (< 45 years), CK levels are elevated, and there is a greater involvement of the proximal muscles of the upper limbs [64]. In a case series, the antibody status against NT5C1A was positive in just under two-thirds of patients [64]. Hepatitis C is considered a risk factor [65].

Another rare manifestation is polymyositis; clinically, it presents with subacute onset of proximal tetraparesis and elevated CK levels.

### Nemaline-myopathy

This is a rare HIV-associated myopathy mainly in the early stages of the infection. Pathophysiologically, it is thought to be caused by the deposition of so-called nemaline rods as a result of an HIV-induced immune response [66]. Clinically, patients present with slowly progressive, proximal, atrophic tetraparesis.

### Myopathy in diffuse infiltrative lymphocytosis syndrome (DILS)

This condition has already been described in the section on “HIV-1-associated neuropathies”. In some cases, patients present with proximal tetraparesis as a sign of muscle involvement.

## Opportunistic infections

Opportunistic cerebral infections are caused by parasites (Toxoplasma gondii), viruses (JC virus, cytomegalovirus [CMV], Varizella zoster (VZV) and Epstein-Barr virus (EBV)), fungi (Cryptococcus neoformans) or bacteria (mycobacteria) in people with HIV or other immunocompromised patients. The incidence of these diseases has decreased significantly as a result of modern cART. The most common opportunistic infections are progressive multifocal leukoencephalopathy (PML) (0.7 per 1,000 patient-years), toxoplasma encephalitis (0.4 per 1,000 patient-years), followed by cryptococcal meningitis (0.2 per 1,000 patient-years) [67–69]. Regional differences account for, e.g., a much higher incidence of tuberculous meningitis in Africa [67–70]. Other risk factors (in addition to a low CD4 cell count) include: high HIV viral load (over 100,000 RNA copies/ml), age > 60 years, resistance to antiretroviral drugs, poor adherence, as well as drug use and alcohol dependence.

### Primary central nervous system lymphoma

Primary central nervous system (CNS) lymphoma is the most common cerebral neoplasm in HIV-infected patients and accounts for 15% of non-Hodgkin’s lymphomas in HIV-positive individuals. These are predominantly immunoblastic, monoclonal B-cell non-Hodgkin’s lymphomas, almost 100% of which are associated with EBV. The prognosis remains poor even today. The median survival time is 2 to 4 months without treatment and 1.5 years with chemotherapy [71, 72].

## Immune reconstitution syndrome (IRIS)

A feared complication in the early stages (mostly < 4 months) of anti-cART is IRIS [72–74], which is characterised by an atypical course of an opportunistic infection. IRIS can manifest as “unmasking IRIS” (an opportunistic infection becoming clinically apparent only after the start of cART) or “paradoxical IRIS” (symptoms of an opportunistic infection recurring after initial improvement under newly initiated cART). Risk factores are active or subclinical opportunistic infection, a CD4 + cell count below 50/µl, a high plasma viral load at the start of cART and a rapid reduction of plasma HIV viral load to below the limit of detection after start of cART.

## Diagnostics

### Aseptic leptomeningitis

#### Mandatory investigations

1. Neurological examination Mostly normal; meningeal signs may be absent
2. CT or MRI scan
3. Lumbar puncture (typical finding of a mononuclear pleocytosis and a high HIV viralload)
4. Detection of HIV infection (PCR mandatory because in this early stage an antibodyresponse may not yet have been mounted)

Neuropathological findings:

Lymphomonocytic infiltrates in the subarachnoid and Virchow-Robin spaces and perivenous inflammatory infiltrates [7, 75].

## HIV-1-associated dementia

The primary aim is to rule out other causes such as opportunistic infections, lymphomas, comorbidities (e.g. syphilis or hepatitis C), metabolic disorders (e.g. vitamin B12 deficiency), age-related neurodegenerative diseases (e.g. Alzheimer’s disease, vascular dementia or Lewy body dementia). The diagnosis of “HAD and its stages” is made clinically.

### Mandatory investigations

1. Neurological examination Is usually normal in stages 1 and 2 of HAND. Early signs at the transition to HAD are often non-specific and include slowed saccadic eye movements, hypomimia, tremor, frontal disinhibition phenomena or slowed fine motor skills, as well as difficulties with tandem gait [29, 76]
2. Neuropsychological screening (e.g. HIV Dementia Scale)
3. Neuropsychological testingCommonly affected domains include: attention, working memory, executive functionsand information processing speed [77]. Patients on long-term cART also frequentlyshow impairments in learning processes and complex attention. Even though the phenotype is variable, comprehensive testing helps both the patient and the clinician toobjectively identify deficits and develop compensatory strategies
4. Rule out depressive pseudodementia

### Tests required in individual cases

#### Blood tests

Immune status: Treatment is rarely initiated by a neurologist, as the majority of people living with HIV are closely monitored by specialist infectious disease clinics and usually undergo routine monitoring of their CD4+ cell count and HIV viral load every 3 to 6 months. Knowledge of the CD4+ cell count is important for the assessment of HAND. The CD4+ cell count is inversely correlated with the likelihood of HAND [29, 78]. This correlation is already evident at values below 350 CD4+ cells/µl.

Lumbar puncture: (including HIV viral load, with resistance testing if necessary, and for differential diagnostic purposes: JC virus and cytomegalovirus PCR)Pleocytosis of up to 20 cells/µl is frequently observed in patients with HAND; cell counts of > 50/µl, however, are rarely reached. A discrepancy between suppressed HIV viral load in plasma and a detectable viral load in CSF indicates local replication (“viral CNS escape”) and requires adjustment of antiretroviral therapy with more CSF-penetrating agents (see chapter “Highly active antiretroviral combination therapy”; CPE score). In cases of viral loads > 1000 copies/ml, resistance testing should always follow. If the patient’s age is consistent with neurodegenerative dementia, the dementia markers tau, phospho tau and Aβ 40/42 should be determined.

#### Cerebral imaging

Magnetic resonance imaging (MRI) is the method of choice. Typical findings include diffuse atrophy with volume loss in the white matter and in cortical and subcortical areas [79]. In addition, hyperintensities in the white matter exceeding age-related norms are frequently observed, which appear to correlate broadly with cognitive impairment, CD4+ cell count and cardiovascular risk profile [80].

#### EEG

There are no EEG changes specific to HIV. A reduction in alpha activity at rest is frequently reported [82]. Severe dementia may be associated with generalized changes. Given the increased prevalence of epilepsy in patients with HAD, the use of EEG is advisable.

Neuropathological findings in advanced cases (overview in [7]):

Macroscopy: general atrophy with secondary enlargement of the ventricular system and atrophy of the deeper nuclear structures, with foci of demyelination and vacuolization

Microscopy:

- Multiple, disseminated, but predominantly subcortical nodular microglial foci
- Macrophages
- Multinucleated giant cells (often paravascular)
- Presence of HIV antigen or specific nucleic acids in monocytes/macrophages and microglial cells, also present to a lesser extent in astrocytes
- Astrogliosis
- Diffuse and regional demyelination (manifestation of an inflammatory disruption of the blood-brain barrier)
- Neuronal cell loss (predominantly in the frontal cortex)

Subtypes:

HIV-1-associated leukoencephalopathy

This encompasses the following entities: vacuolar encephalopathy; progressive diffuse or diffuse inflammatory leukoencephalopathy; and HIV-associated demyelination, whereby different forms may coexist in a single patient [83].

## HIV-1-associated myelopathy

### Mandatory investigations

1. Somatosensory and motor evoked potentials provide objective evidence of the extent of spinal cord involvement.
2. Electroneurography to rule out concomitant polyneuropathy
3. Spinal MRI to rule out mechanical spinal cord compression; possible findings: Atrophy of the spinal cord, usually in the thoracic and/or cervical regions
4. Laboratory measurement of vitamin B12, holotranscobalamin or methylmalonic acid to rule out funicular myelopathy
5. Lumbar puncture and CSF analysis to rule out viral myelitis caused by CMV, HTLV-1, HSV and VZV (serology or PCR), toxoplasmosis, syphilis and lymphoma

## HIV-1- associated neuropathies

### Mandatory investigations

1. Medical history (focus on immune status and non-HIV-1-associated risk factors for PNP).
2. Medication history (focus on neurotoxic medications).
3. Neurological examination findings.
4. Extended basic laboratory tests, with particular attention to glucose metabolism (HbA1c, OGTT if necessary), vitamin B12 and folic acid levels, and, where appropriate, vasculitis parameters and pathogen serology (CMV, VZV, EBV, HSV, hepatitis C, Treponema pallidum, Borrelia)
5. Nerve conduction studies

### Tests required in individual cases

#### Cerebrospinal fluid analysis

In patients with AIDP or CIDP, a albuminocytologic dissociation is observed, as in the non-HIV-associated forms; however, unlike in non-HIV associated cases mild. lymphomonocytic pleocytosis is frequently found [84, 85].

#### Electromyography

*SEP* to rule out HIVM.

*Morphological examination for small-fibre neuropathy* (e.g. skin punch biopsy, corneal. confocal microscopy)

*Functional testing for small-fibre dysfunction* (e.g. quantitative sensory testing). *MRI of the lumbar spine* with contrast agent (Question: increased contrast.enhancement in the nerve roots in HIV-associated, atypical CIDP? )

*Nerve biopsy* (possibly combined muscle and nerve biopsy).

## HIV-1-associated myopathies

### Mandatory investigations

1. Medical history, with particular attention to antiretroviral therapy (zidovudine).
2. Laboratory tests to detect elevated levels of CK and/or myoglobin.
3. Electromyography to detect myopathic changes.

### Tests required in individual cases

*Myositis-specific or myositis-associated autoantibodies*. (e.g. NT5C1A in cases of suspected HIV-associated inclusion body myositis) *Muscle MRI*.

*Muscle biopsy* for histopathological detection of inflammation, patterns of mitochondrial damage or to classify a myositis entity (e.g. inclusion body myositis). Additional *cardiac and pulmonary diagnostics* to assess organ involvement and establish a differential diagnosis (e.g. in cases of suspected myositis).

## Opportunistic cerebral infections

### Mandatory investigations

1. Neurological examination.
2. Fever chart (for Toxoplasma gondii, cryptococcal meningitis and mycobacteria).
3. Cranial CT with contrast medium:
  - Toxoplasma gondii, ring-shaped contrast-enhancing, usually multiple lesions with perifocal oedema. Radiologically, cerebral toxoplasmosis cannot be reliably distinguished from primary CNS lymphoma.
  - Cryptococcus neoformans meningoencephalitis, possibly diffuse cerebral oedema.
  - JC virus infection, multifocal hypodense lesions in the parenchyma, usually without contrast enhancement.

4. Cranial MRI (T1- and T2-weighted sequences as well as T1-weighted sequences with contrast):
  - Toxoplasma gondii, ring-shaped contrast-enhancing, usually multiple lesions with perifocal oedema; MRI is significantly more sensitive than CT. Radiologically, cerebral toxoplasmosis cannot be reliably distinguished from primary CNS lymphoma even on MRI. In SWI, haemorrhagic changes are frequently seen within the lesion.
  - JC virus, uni- or often multifocal hyperintensities in the white matter on T2- and FLAIR-weighted images with little or no contrast enhancement.
  - Cytomegalovirus infection, possibly multifocal punctate areas of increased signal intensity on T2-weighted images; in some cases, tumour-like lesions and ventriculitis with periventricular and ependymal gadolinium enhancement are also possible.
  - Cryptococcal infection, possibly meningeal enhancement; rarely, focal intracerebral lesions with ring-shaped contrast enhancement, neuroimaging is often unconspicuous.
  - Mycobacterial infections (meningeal contrast enhancement, microabscesses with ring-shaped contrast enhancement).

5. Lumbar puncture:
  - CSF pressure measurement: In cryptococcal meningitis, CSF pressure is usually significantly elevated.
  - Microscopy: Cell count, bacterial and mycobacterial staining, and India ink smear for cryptococcosis in fresh CSF (no more than one hour old).
  - Pathogen detection:
    i. Cultures: bacteria, mycobacteria, fungi.
    ii. Multiplex PCR to detect, among others, CMV, HSV, VZV, Cryptococcus,
    iii. JC virus PCR: diagnostic when supported by relevant clinical and radiological findings and performed in reliable laboratories. Repeat if result is negative but suspicion persists
    iv. Toxoplasma gondii PCR: low sensitivity (depending on the study < 50%), high specificity, but often helpful in conjunction with radiology and clinical findings, positive Serology
    v. EBV PCR, associated with CNS lymphoma
    vi. PCR for tuberculosis: low sensitivity, but high specificity

6. Serology:
  - Latex antigen test for cerebral cryptococcosis. The cryptococcal antigen in serum is more sensitive than antigen testing in cerebrospinal fluid or the ink smear.
  - Syphilis serology (FTA-IgG and FTA-IgM tests, VDRL, TPHA).
  - Toxoplasma gondii.

High prevalence in the general population – therefore IgG is often positive even in the absence of clinical symptoms. In the case of focal, contrast-enhancing lesions, a positive IgG serology is sufficient for a suspected diagnosis and to initiate specific treatment. IgM testing is not helpful, as this represents reactivation rather than new infections. Even with completely negative toxoplasmosis serology, cerebral toxoplasmosis cannot be ruled out.

#### Tests required in individual cases

Brain biopsy (for Toxoplasma gondii if trial of antiparasitic treatment fails after 2–3 weeks; for suspected JC virus infection if a repeat cerebrospinal fluid PCR test is negative).

## Primary CNS lymphoma

### Mandatory investigations

1. Neurological examination.
2. MRI with contrast (ring-shaped, contrast-enhancing, unilocular or multilocular masses). Radiologically, primary CNS lymphoma cannot be reliably distinguished from cerebral toxoplasmosis.
3. Lumbar puncture (including EBV PCR and cytology).
4. “Staging” to rule out secondary lymphoma (CT of the abdomen and thorax, palpation and ultrasound of lymph node regions and testes, Yamshidi puncture if the patient’s general condition permits, ophthalmological consultation).
5. If lymphoma is strongly suspected, a brain biopsy to confirm the diagnosis before starting treatment.

## Immune reconstitution syndrome (IRIS)

### Mandatory investigations

1. Neurological examination, vital signs.
2. Cranial MRI.
3. Depending on the suspected opportunistic cerebral infection or in the case of lymphoma, proceed as described there. Note: As a result of an excessive immune response in IRIS, pathogen detection may be more difficult than in an OI without IRIS.
4. If vasculitis is suspected, perform MR angiography
5. If optic neuritis is suspected, consult an ophthalmologist and perform VEP

## Treatment

### HIV-1-associated dementia and its prodromal stages

Initiation of highly active antiretroviral therapy (cART) that is effective based on resistance testing. For pathogenetic reasons, substances that cross the blood-brain barrier as effectively as possible (CPE score, see below) should be used. However, in the prophylaxis of HAD, cART – even if it is able to cross the blood-brain barrier–is not always successful [86]. In the presence of depression, antidepressant medication should be administered, taking into account pharmacokinetic interactions.

## Recommendations

### Highly active antiretroviral combination therapy (cART)

Since 1996, cART has been used with the aim of achieving the most effective suppression of plasma viral load possible. The basic principle of cART is the inhibition of viral replication via various mechanisms. Nowadays, first-line therapy is predominantly administered in combination with an integrase inhibitor. Treatment should be initiated in consultation with an HIV specialist or an infectious disease specialist.

The CNS acts as a separate compartment for the HIV virus and the virus can evade suppression there, particularly when cART has unfavourable pharmacokinetic properties. The CSF penetration of antiretroviral drugs is characterised by the CNS penetration efficacy score (CPE score) [87], which ranges from 1 (no or low penetration) to 4 (highest penetration). The concentration of antiretroviral substances in the CSF is not equivalent to their intraparenchymal concentration. However, according to initial data, this is comparable for the majority of substances [88]. Efavirenz, on the other hand, appears to accumulate in the brain parenchyma and significantly exceed the CSF concentration [89], which is likely to be the cause of the commonly observed central side effects. Lamivudine, which is assigned a CPE score of only 2, also demonstrated high penetration into cerebrospinal fluid in a recent study [90]. Although the correlation between the CPE score and neurocognitive characteristics is a matter of debate, this information appears particularly relevant in patients with very high CSF viral loads. Further developments of the CPE score include the ability of antiretroviral agents to control HIV infection in monocytes and macrophages (monocyte efficacy score) [91], CNS-specific resistance behaviour [92] and pharmacokinetic modelling [93]. However, these have not yet found application in everyday clinical practice.

Future research strategies will focus on antiretroviral therapies capable of suppressing viral replication in myeloid cell lines in the CNS, for example through the use of nanoformulated antiretroviral therapies (nanoART) [94, 95].

Table 1 provides an overview of the antiretroviral agents currently available, including their ability to cross the blood-brain barrier, as assessed using the CPE score (modified from [96, 97]). Numerous agents are available as combination preparations. Agents in italics are no longer of clinical significance in Germany. In Switzerland, too, these play a significantly minor role; by contrast, treatment with efavirenz and nevirapine is continued in patients who tolerate the drug well.

**Table 1:** Overview of antiretroviral agents and their CNS penetration (CPE score), adapted from [96, 97]

	Abbreviation	Year of approval	CPE-Score
Nucleoside reverse transcriptase inhibitors (NRTIs)			
Azidothymidine/Zidovudine	AZT/ZDV	1987	4
* Didanosine*	ddI	1991	2
* Stavudine*	d4T	1994	2
Lamivudine	3TC	1995	2
Abacavir	ABC	1998	3
Tenofovir disoproxil fumarate	TDF	2001	1
Emtricitabine	FTC	2003	3
Tenofovir alafenamide	TAF	2015	1
Non-nucleoside reverse transcriptase inhibitors (NNRTIs)			
* Nevirapine*	NVP	1996	4
* Delavirdine*	DLV	1997	3
* Efavirenz*	EFV	1998	3
Etravirine	ETR	2008	2
Rilpivirine	RPV	2011	2
Doravirine	DOR	2018	/
Protease inhibitors (PI)			
* Saquinavir mesylate*	SQV	1995	1
Ritonavir	RTV	1996	1
Indinavir	IDV	1996	3
Nelfinavir	NFV	1997	1
* Lopinavir*	LPV	2000	3
Atazanavir sulphate	ATV	2003	2
* Fosamprenavir calcium*	FOS	2003	2
* Tipranavir*	TPV	2005	1
Darunavir	DRV	2006	3
Fusion inhibitor			
Enfuvirtide	T-20	2003	1
CCR5 inhibitors			
Maraviroc	MVC	2007	3
Integrase inhibitors (INIs)			
Raltegravir	RAL	2007	3
Dolutegravir	DTG	2013	4
Elvitegravir	EVG	2014	2
Bictegravir	BIC	2018	/
Cabotegravir	CAB	2021	/
Monoclonal CD4 antibodies/post-attachment inhibitors (PAI)			
Ibalizumab	IBA	2018	/
Pharmacokinetic boosters			
Ritonavir	RTV	1996	1
Cobicistat	COBI	2014	0

Explanation: / = No data available

#### Indications

All HIV infected patients should be treated with cART. A possible exception are the rare non-progressors with a stable CD4 cell count and very low viral load. However, in cases of potential HIV-related neurological symptoms, cART should also be initiated in these patients.

#### Interactions

The spectrum of interactions involving antiretroviral substances is complex. For specific details, the reader is referred to the following website: http://www.hiv-druginteractions.org/. Interactions should be checked before any new drugs are used.

Of particular note is the interaction between cART and antiepileptic drugs, which, due to the frequency of epileptic seizures in HIV-infected individuals (up to 11% [99]), is of particular importance in neurological practice [98, 100–102]. Before administering an antiepileptic drug, particularly valproic acid, phenytoin, lamotrigine and carbamazepine, http://www.hiv-druginteractions.org/ should be consulted.

### HIV-1-associated myelopathy

No specific treatment has been validated by clinical trials (only case reports). cART should be initiated. If detectable HIV viraemia persists whilst on cART, the cART regimen should be changed.

### HIV-1- associated neuropathies

Treatment involves both causal (Table 2) and symptomatic approaches.

**Table 2:** Causal treatment approaches are available for:

HIV-DSP	cART, where possible, excluding potentially neurotoxic substances Improvement in pain symptoms through cART [103]
Antiretroviral-induced neuropathy	Discontinuation of the toxic substance in consultation with the HIV specialist.
Acute inflammatory demyelinating polyradiculoneuritis (GBS)	Immunoglobulins, plasmapheresis or immunoadsorption (see treatment for HIV-seronegative patients) cART, where possible, excluding potentially neurotoxic substances
Chronic inflammatory demyelinating polyradiculoneuropathy (CIDP)	Corticosteroids [84] or immunoglobulins (see treatment for HIV-seronegative patients) Note the interaction between cART and corticosteroids (http://www.hiv-druginteractions.org/) Caution: Increased risk of avascular necrosis of the femoral head or osteoporosis with steroids Where possible, cART should exclude potentially neurotoxic substances
Vasculitic polyneuropathy	Treatment is based on the DGN guideline taking into account the clinical findings and the underlying immunodeficiency. (LL_Therapie_akuter_und_chronischer_immunvermittelter_Neuropathien_und_Neuritiden_2018) Steroids are frequently used, e.g. prednisone 100 mg/day for 2–3 weeks followed by tapering. Note the interaction between cART and corticosteroids (http://www.hiv-druginteractions.org/). Caution: Increased risk of avascular necrosis of the femoral head or osteoporosis with steroids. Where possible, cART should exclude potentially neurotoxic substances
Polyneuropathy in diffuse infiltrative lymphocytosis syndrome	The condition responds well to cART combined with steroid therapy [58, 104]. Note the interaction between cART and corticosteroids (http://www.hiv-druginteractions.org/)
Polyradiculoneuritis caused by opportunistic pathogens	Pathogen-specific treatment

The management of HIV-associated neuropathic pain focuses primarily on symptomatic pain relief with the aim of improving quality of life. The majority of the medications used are off-label and include substances from the groups of anticonvulsants, antidepressants and non-specific analgesics such as opiates, as well as topical agents [45]. In a meta-analysis of prospective, double-blind, randomised controlled trials, only high-dose capsaicin, cannabis and recombinant nerve growth factor were shown to be superior to placebo [105]. The local application of an 8% capsaicin patch led to a significant reduction in pain lasting 12 weeks in patients with HIV-associated DSP, compared with a control group treated with a low dose (capsaicin 0.1%) [106]. In a randomised, placebo-controlled study, a significant reduction in pain intensity and, secondarily, in sleep disturbances was reported with gabapentin [107]. In two placebo-controlled studies, pregabalin was not shown to be superior to placebo [108, 109]. For amitriptyline, three randomised controlled trials showed no therapeutic effect, regardless of concomitant antiretroviral therapy [110–112].

### HIV-1-associated myopathies

Mild cases involving myalgia alone are usually adequately treated with NSAIDs.

Antiretroviral therapy is usually indicated. Only in cases of toxic myopathies, such as AZT myopathy, is discontinuation or switching of the medication the treatment of choice. Symptom resolution may take approximately 4–6 weeks.

If discontinuation does not lead to improvement, a trial of prednisone (see above), as indicated for polymyositis, is advisable.

Of the inflammatory myositis conditions, HIV-associated polymyositis can usually be treated effectively with prednisone (100 mg/day for 3–4 weeks, then tapered off slowly; alternatively, pulse steroid therapy). For HIV-associated inclusion body myositis, intravenous immunoglobulins (0.4 g/kg body weight daily for 5 days) may be used off-label, analogous to non-HIV-associated IBM.

Nemaline myopathy and myopathy occurring in the context of diffuse infiltrative lymphocytosis syndrome also respond well to the administration of prednisone (see above for dosage).

### Opportunistic CNS diseases

Dosage recommendations vary significantly depending on the publication and guideline. This guideline is based on the current EACS Guidelines (https://eacs.sanfordguide.com/en/eacs-hiv/eacs-section4/ois/).

### Toxoplasma gondii infection

- Pyrimethamine p.o. (200 mg on day 1; from day 2 onwards, 1 x 75 mg for body weight ≥ 60 kg; 1 x 50 mg for body weight < 60 kg) +
- Sulfadiazine p. o. (≥ 60 kg body weight 2 x 3000 mg/d; < 60 kg body weight 2 x 2000 mg/d)
- + folic acid p. o. (15 mg/d) [72, 113-116]
- In case of sulphonamide intolerance: clindamycin (4 x 600 mg/day p.o.) + pyrimethamine (as above)
- The second-line treatment is monotherapy with trimethoprim (TMP)/sulfamethoxazole (SMX) p.o. (2 x 5 mg TMP/kg body weight/day + 2 x 25 mg SMX/kg body weight)
- Further alternatives:
  - Azithromycin p.o. (1 x 0.5–1 g/day) together with pyrimethamine and folic acid; atovaquone p.o. (2 x 1500 mg/day) together with pyrimethamine and folic acid or sulphadiazine (as above)

- In patients with malabsorption or severe diarrhoea, treatment may be switched to intravenous cotrimoxazole 5 mg TMP/kg body weight twice daily, or, in cases of sulphonamide allergy, to clindamycin 4 x 600 mg intravenously + pyrimethamine and folic acid

Following the initial course of treatment lasting approximately 6 weeks, maintenance therapy is required, for example with sulphadiazine (1 g twice daily) and pyrimethamine (25 mg daily) plus folic acid (7.5 mg daily). This maintenance therapy may be discontinued under clinical supervision if there is an optimal response to antiparasitic therapy and successful antiretroviral treatment with immune reconstitution (CD4 > 200 for > 3–6 months and undetectable viral load).

### JC-Virus-Infection (PML)

- cART: Immune reconstitution often leads to partial remission and stabilisation, in some cases lasting for months or even years. There is no specific therapy proven to be effective. Under cART, immune reconstitution syndrome (IRIS) may initially occur, with clinical deterioration and contrast-enhancing lesions. In cases of severe IRIS, steroids may be considered.
- Newer drugs: Recent case series have demonstrated the potential efficacy of the checkpoint inhibitors pembrolizumab and nivolumab [117] and the CCR5-blocking antiretroviral substance maraviroc [118]. However, these compounds need to be studied in randomised controlled trials.
- A new therapeutic approach involves the use of allogeneic virus-specific T cells [119].

### Cytomegalovirus infection of the CNS

- Standard treatment: Ganciclovir i.v. (2 × 5 mg/kg body weight/day) or Foscarnet i.v. (2 × 90 mg/kg body weight/day). Dosage should be adjusted according to renal function.
- Following induction therapy, maintenance therapy with a reduced dose (foscarnet 90 mg/kg body weight/day + ganciclovir 5 mg/kg body weight/day) should be administered.
- Alternatively: Cidofovir i.v. (1 × 5 mg/kg body weight/week) for at least 3 weeks if standard therapy is not tolerated.

### Cryptococcal meningitis

- Induction therapy: liposomal amphotericin B 3 mg/kg body weight plus flucytosine (100 mg/kg body weight per day, divided into 4 doses) over 2 weeks [68-70]
  - Recently, a single dose of liposomal amphotericin B 10 mg/kg body weight with additional doses of flucytosine (100 mg/kg body weight/day, divided into 4 doses) and fluconazole (1200 mg four times daily for 14 days) has also been recommended as an alternative (https://eacs.sanfordguide.com/en/eacs-hiv/eacs-section4/ois/cryptococcosis)
  - Alternatively, the combination of amphotericin B (0.7–1.0 mg/kg body weight/day) plus flucytosine (100 mg/kg body weight/day) may be considered.
  - Alternatives for induction therapy include amphotericin B plus fluconazole, fluconazole plus flucytosine, and fluconazole and itraconazole as monotherapy. However, the effectiveness of these therapies is considered to be lower than that of standard therapy.

- This is followed by consolidation with fluconazole (400 mg/day orally) for 8 weeks and maintenance therapy with a reduced dose of fluconazole (200 mg/day) for at least 1 year.
- If cART is successful, treatment may be discontinued once the CD4 cell count has risen to > 100 cells/µl and the viral load has been low or undetectable for at least 3 months.

### Tuberculosis of the CNS

The following remarks do not apply to atypical mycobacteria, which, however, very rarely cause CNS infections.

- Initial treatment for 2 months: four-drug combination:

INH oral (3–5 mg/kg body weight/day, maximum daily dose 300 mg) with pyridoxine (vitamin B6) (20–60 mg/day) +.

rifampicin oral (600 mg/day) +.

Ethambutol p.o. (20–25 mg/kg body weight/day) +.

Pyrazinamide p.o. (15–30 mg/kg body weight/day, maximum 2000 mg/day)

- Treatment adjustment based on resistance testing. In case of resistance, consult specialists.
- After 2 months: dual or triple combination therapy for up to a total duration of 12 months (possibly longer in cases of persistent disease): INH p.o. (3–5 mg/kg body weight/day, maximum daily dose 300 mg) + rifampicin p.o. (600 mg/day)
- Directly observed therapy (DOT) recommended, including, if necessary, a switch to 3-times-weekly therapy: INH p.o. (15 mg/kg body weight/day, maximum daily dose 900 mg) + rifampicin p.o. (600 mg/day)
- Concomitant medication: Vitamin B6 (20–60 mg/day) for INH-associated polyneuropathy, possibly allopurinol 300 mg/day for pyrazinamide-induced hyperuricaemia; ophthalmological examinations due to possible ethambutol opticopathy.

Cave.

- Interactions between rifampicin and antiretroviral therapy are very common (protease inhibitors, non-nucleoside reverse transcriptase inhibitors, integrase inhibitors); if necessary rifampicin should be replaced by rifabutin.
- IRIS occurs very frequently in cases of CNS tuberculosis. Antiretroviral therapy should not be started at the same time as TB treatment, but only after at least two weeks. If the CD4 cell count is below 100/µl, consider pre-emptive corticosteroids (prednisone 40 mg orally for 2 weeks, followed by 20 mg for 2 weeks) when starting cART.

### Neurosyphilis

For information on neurosyphilis, please refer to the relevant guideline [120]. The main deviation from the standard management in non-HIV patients is the reduced reliability of the VDRL test as a marker of disease activity in HIV infection. This is because HIV does not only suppress but also modulate the immune system of HIV patients which may result in active neurosyphilis being present despite negative VDRL test. Therefore, in the presence of clinical symptoms (prolonged headaches, cranial nerve palsies – cranial nerves VII and VIII are frequently affected), positive syphilis reactions in serum, possibly with rising titres at follow-up examinations, as well as a positive VDRL reaction and an inflammatory CSF syndrome with a positive FTA-Abs, treatment should be administered consistently with 6 × 4 million IU penicillin G daily for 14 days or 2 g ceftriaxone intravenously in cases of penicillin allergy. Whether a lumbar puncture should be performed in cases of late latent syphilis without neurological symptoms is a matter of debate. Some authors recommend this procedure if the VDRL titre is ≥ 1:32 or CD4 cell count is below 350 cells/µl [121].

### Primary CNS lymphoma

- cART: Immunoreconstitution alone can lead to a significant prolongation of median survival.
- Radiation: 30–60 Gy, whole neurocranium; improves the prognosis only slightly in patients in good general health.
- Chemotherapy: Methotrexate (3 g/m^2^) administered systemically every 14 days or (if the patient is in good general health) polychemotherapy (vincristine, procarbazine and lomustine) prolongs median survival by approximately 12 months.
- Antiviral agents: ganciclovir (sometimes in combination with IL-2) or hydroxyurea – single cases of remission have been reported.

### Immune reconstitution syndrome (IRIS)

The best approach to managing IRIS is to continue cART and treat any complications that arise. The use of corticosteroids is a matter of debate; whilst they can be life-saving, they can also weaken the immune system once again.

## Data Availability

No datasets were generated or analysed during the current study.
